# Rapid Diagnosis of *Mycobacterium marinum* Infection by Next-Generation Sequencing: A Case Report

**DOI:** 10.3389/fmed.2022.824122

**Published:** 2022-02-04

**Authors:** Fanfan Xing, Simon K. F. Lo, Yuanchao Ma, Jonathan Daniel Ip, Wan-Mui Chan, Meixun Zhou, Miaozi Gong, Susanna K. P. Lau, Patrick C. Y. Woo

**Affiliations:** ^1^Department of Clinical Microbiology and Infection Control, The University of Hong Kong - Shenzhen Hospital, Shenzhen, China; ^2^Department of Microbiology, Li Ka Shing Faculty of Medicine, The University of Hong Kong, Pokfulam, Hong Kong SAR, China; ^3^Department of Pathology, The University of Hong Kong - Shenzhen Hospital, Shenzhen, China

**Keywords:** *Mycobacterium marinum*, non-tuberculosis mycobacteria, next-generation sequencing, Oxford Nanopore, MinION

## Abstract

We present the first report of histology- and culture-proven *Mycobacterium marinum* infection diagnosed by next-generation sequencing (NGS). It took <2 days to make a microbiological diagnosis using the Oxford Nanopore Technologies' MinION device, compared to 20 days for the mycobacterium to be isolated from the tissue biopsy. NGS is particularly useful for culture-negative and slow-growing microorganism infections, such as mycobacterial, fungal and partially treated pyogenic bacterial infections. Due to its low equipment cost, short turn-around-time and portable size, the Oxford Nanopore Technologies' MinION device is a useful platform for NGS in routine clinical microbiology laboratories.

## Introduction

*Mycobacterium marinum* is a pigmented slow-growing non-tuberculous mycobacterium associated with skin and soft tissue infections. The bacterium is commonly found in both fresh or saltwater in many parts of the world (1). Most infections develop 2–3 weeks after direct or indirect contact with contaminated water or fish as papules or ulcerations on the hands and arms, which may progress to ascending lesions. As the bacterium grows optimally at 28–30°C, it is not associated with deep-seated infections.

Traditionally, *M. marinum* infections were diagnosed in the laboratory by culturing and identifying the bacterium as well as histology. As the bacterium is slow-growing, it often takes 2–3 weeks, and in our experience, sometimes up to 6 weeks, to isolate it from the clinical specimen, and a few more weeks for identifying it to the species level. Therefore, methods for rapid identification of the bacterium are crucial for helping the clinicians to start the specific anti-mycobacterial regimen on a timely basis. In this article, we report the first case of *M. marinum* diagnosed by the Oxford Nanopore Technologies' MinION device, a handy platform of next-generation sequencing (NGS) that can be used in routine clinical microbiology laboratories.

## Case Description

**Figure 1:**
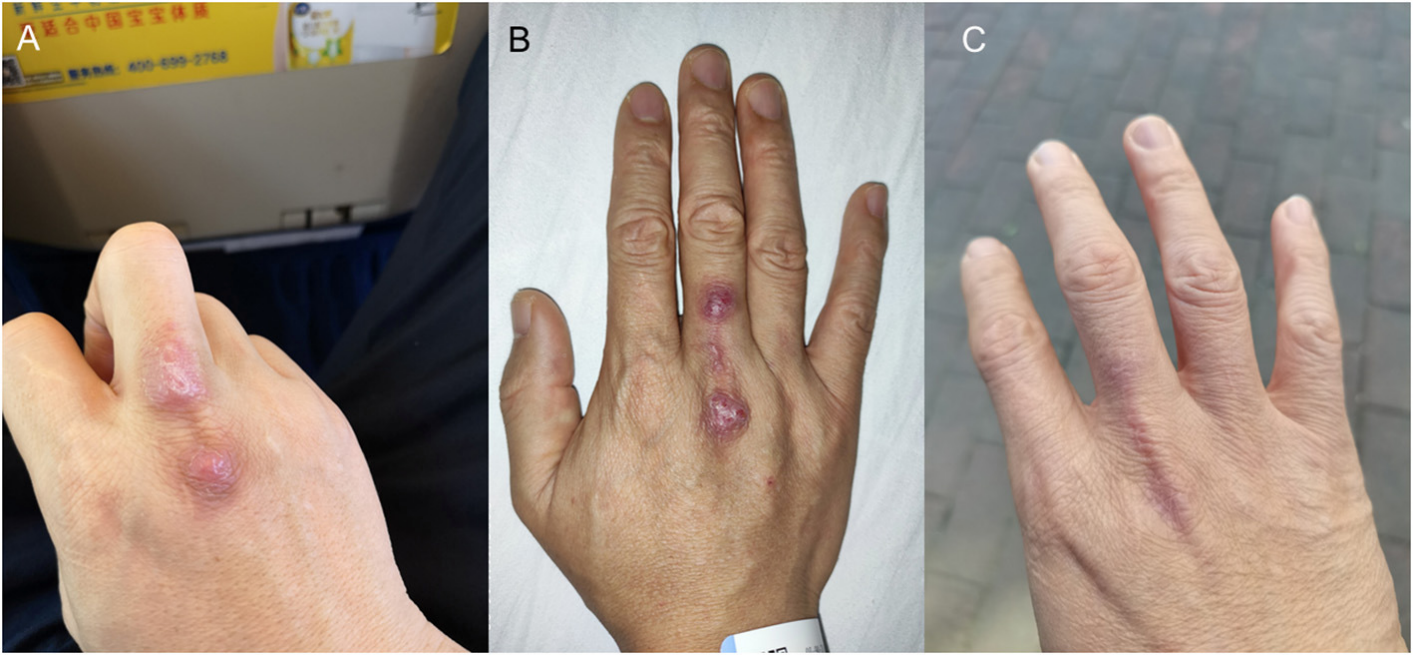
Photos of the patient's right hand. **(A)** Poorly healed primary wound and secondary lesion at the back of the right hand. **(B)** Ulcerated lesions at the back of the right hand. **(C)** Healed wounds after 9 months of treatment.

A 38-year-old Chinese man was admitted because of unhealed wound at the right hand in August 2020. The patient was the owner of a seafood market in Shenzhen, China. Four months before admission, his right middle finger was bitten by a grouper. Topical disinfection was performed. In the next 4 months, he started to recognize small masses developing adjacent to the wound at the back of the right hand as well as in the right elbow and right arm. There was no fever or other systematic symptoms. When the patient was admitted, examination showed the poorly healed primary wound at the back of the right middle finger and a secondary lesion at the back of his right hand (Figure 1A), as well as a 5 × 6 cm subcutaneous nodule at the right elbow and 2 supratrochlear lymph nodes of 1 cm in diameter. The total white cell count was 4.89 × 10^9^/L (normal range, 3.89–9.93 × 10^9^/L) with normal differential count. Liver and renal function tests were normal. C-reactive protein was 1.23 mg/L (normal range, 0–5 mg/L). The subcutaneous nodule and overlying skin at the right elbow were excised for histology and bacterial, fungal and mycobacterial culture. Histological examination revealed chronic granulomatous inflammation but all the culture results were negative. No antibiotic was prescribed.

**Figure 2:**
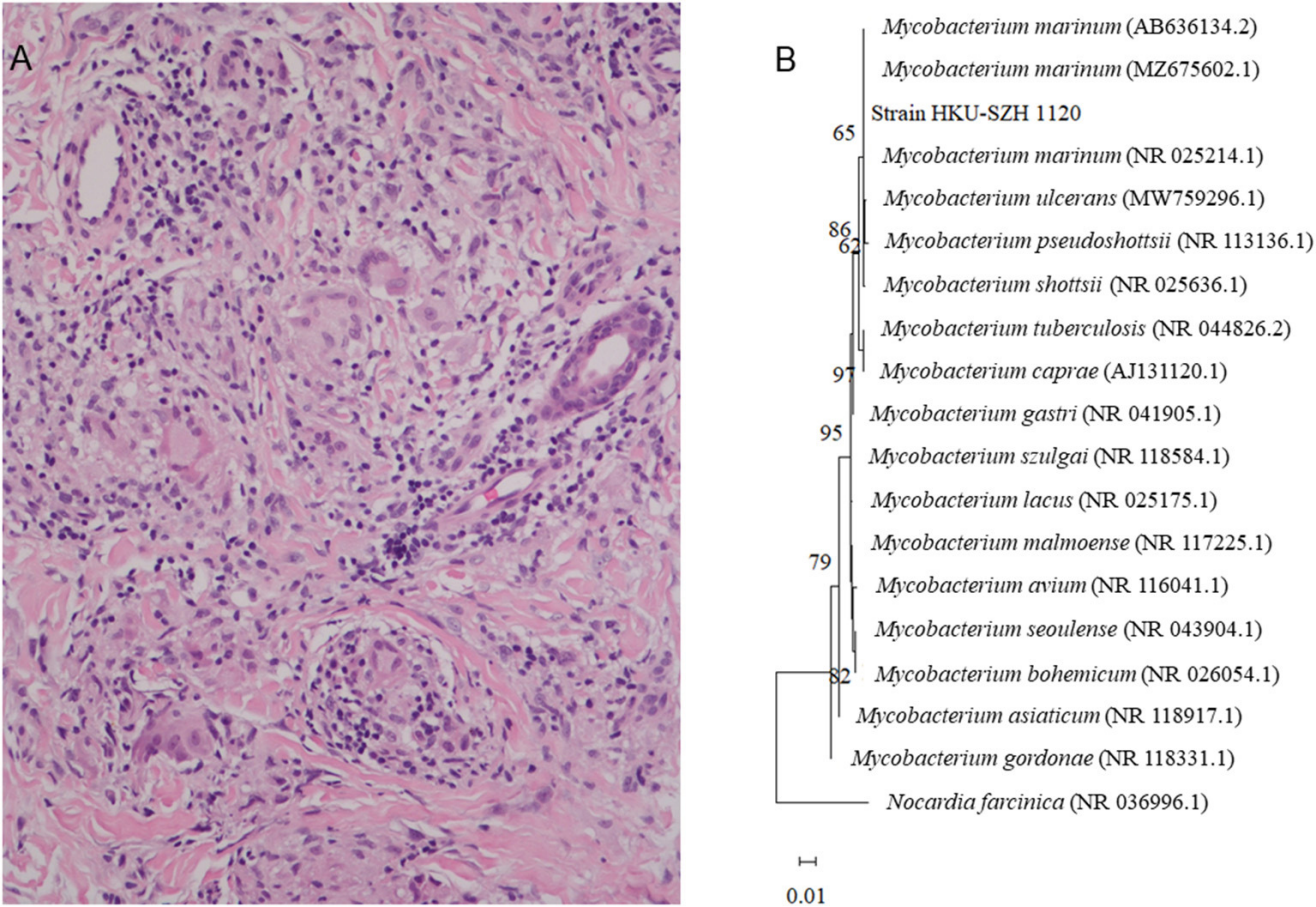
**(A)** Histological section of the dermis, showing granuloma with Langerhans multinucleated giant cells aggregation with surrounding infiltration of lymphocytes (H&E ×200). **(B)** Phylogenetic tree showing the relationships among Strain_HKU-SZH_1120 and other closely related *Mycobacterium* species. A total of 1,231 nucleotide positions in each 16S rRNA gene were included in the analysis. The tree was constructed using the Maximum Likelihood method and Tamura 3-parameter model and rooted using *Norcardia facinica* (NR 036996.1). The bootstrap values calculated from 1,000 trees were shown when they were >60%. The scale bar indicates the estimated number of substitutions per 100 bases. The names and accession numbers (in parentheses) were presented as cited in the GenBank database.

**Figure 3:**
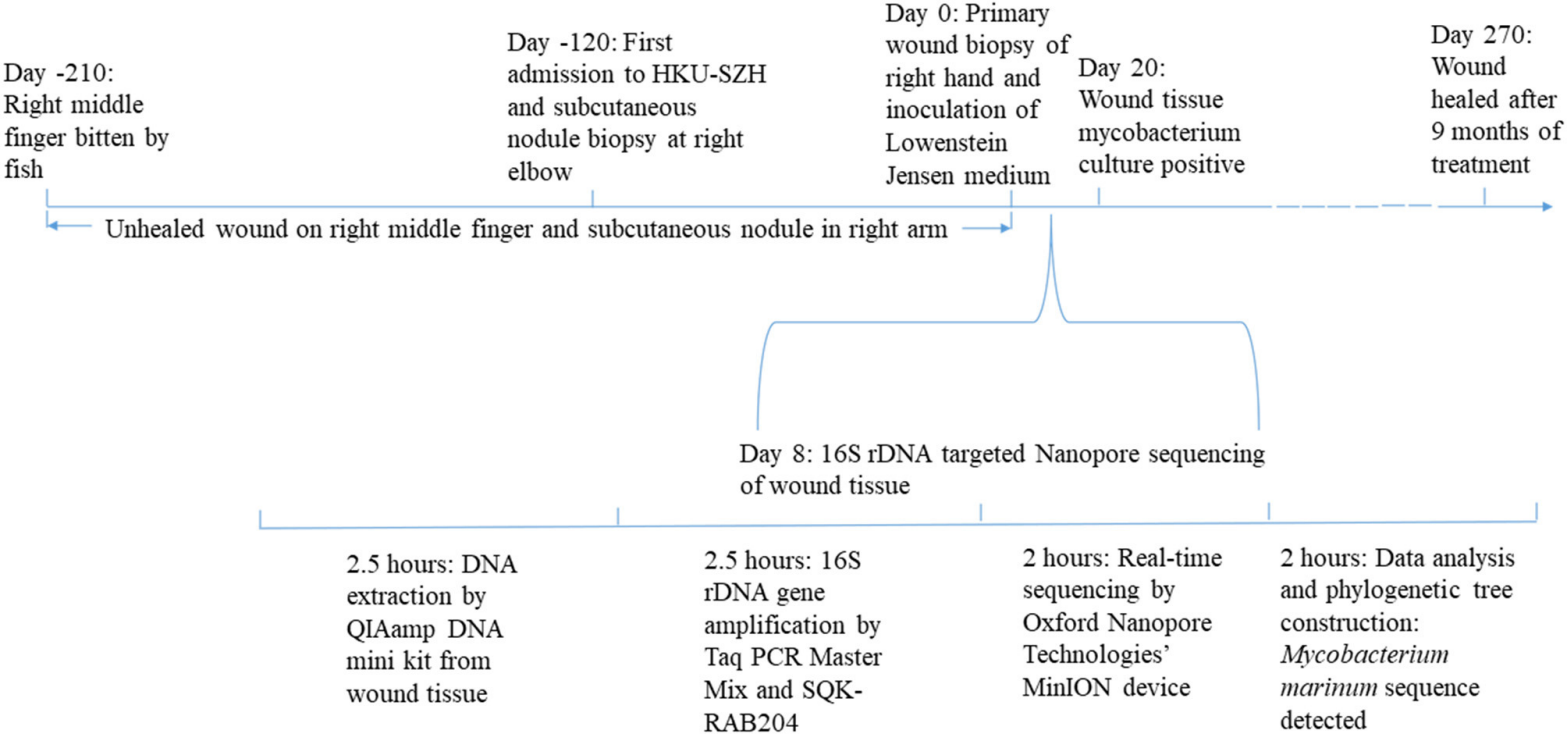
Time line comparing laboratory diagnosis by conventional culture and NGS.

From August to November, the wound on the right middle finger and hand did not heal well and another new painful nodule gradually developed at the right elbow. Examination revealed 2 ulcerated lesions at the back of the right hand (Figure 1B), multiple subcutaneous nodules at the right elbow and palpable supratrochlear, right axillary and cervical lymph nodes. The total white cell count was 3.61 × 10^9^/L (normal range, 3.89–9.93 × 10^9^/L). Liver and renal function tests were normal. C-reactive protein was 17.44 mg/L (normal range, 0–5 mg/L). A biopsy of the primary wound on the right hand was performed. Histological examination showed acanthosis cell layer hyperplasia, granular layer thickening with mild hyperkeratosis and parakeratosis, lymphocytic infiltration in epithelium, proliferation of small vessels in the corium layer with lymphocytic and plasmocytic infiltration, epithelioid cells and Langerhans multinucleated giant cells around appendages in middle and deep dermis but no necrosis and vasculitis (Figure 2A). Ziehl-Neelsen stain was negative. 16S rDNA gene targeted NGS of the tissue samples using the Oxford Nanopore Technologies' MinION device revealed sequences of *M. marinum* (Figure 2B). Mycobacterial culture using Lowenstein Jensen medium was positive with yellow colonies (Strain_HKU-SZH_1120) after 20 days of incubation (Figure 3). Matrix-assisted laser desorption ionization time-of-flight mass spectrometry (MALDI-TOF MS) (top match score 2.283) and 16S rDNA gene sequencing confirmed the identification as *M. marinum*. MALDI-TOF MS was performed by the direct transfer method using the MALDI-TOF MS spectrometer (Bruker Daltonik) and the spectrum was analyzed with IVD MALDI Biotyper 2.3 and reference Mycobacteria library 6.0 (Bruker Daltonik) (2). 16S rDNA gene sequencing was performed according to our previous publications (3, 4). Oral rifampin, minocycline and clarithromycin were prescribed for a total of 9 months. All the wounds were healed and the infection has remained in remission at the time of writing, 2 months from stopping the antibiotics (Figure 1C).

## Discussion

When NGS technologies first appeared in the market, they were mainly used for genome sequencing (5). With the advancement of sequencing chemistries and computational capacity, NGS technologies have matured into clinical applications in the recent years (6). In the clinical setting for infectious diseases, NGS is used most often for patients who have fever without localizing features or culture-negative infections (7, 8). In addition, it is also useful for rapid diagnosis of infections caused by slow-growing microorganisms. For example, we have recently reported its usefulness in rapid diagnosis of fungal infections (9). As for mycobacteria, NGS has been used for the diagnosis of tuberculosis and a number of non-tuberculous mycobacterial infections (10–15). Since many mycobacteria, such as *M. chimaera, M. yongonense, M. avium complex, M. kubicae, M. leprae*, and in the present case, *M. marinum*, are slow-growing or non-cultivable in artificial medium and the anti-mycobacterial therapeutic regimen for different mycobacteria are radically different, it is of crucial importance to identify the mycobacteria involved to the species level in a timely basis in order to prescribe the specific and effective medications for the treatment of the infections. Furthermore, early diagnosis can also avoid unnecessary investigations and side effects resulting from inappropriate medications and hence reduce the related costs. In our experience, it usually takes around 6–12 weeks to isolate and identify a slow-growing mycobacterium from clinical specimens using conventional methods. On the other hand, it will only take up to a few days, even if the samples are sent out to private laboratories, if NGS technology is used. Therefore, this has undeniably important impact in the management of mycobacterial infections.

The Oxford Nanopore Technologies' MinION device is becoming a useful platform for NGS in routine clinical microbiology laboratories. When NGS was first used for laboratory diagnosis of infectious disease, usually more than 10 samples were processed in one sequencing run in order to reduce the sequencing cost per sample. However, the recent invention of Oxford Nanopore Technologies' MinION device has expedited the use of NGS in laboratory diagnosis, due to its low equipment cost, short turn-around-time and portable size (16, 17). When this technology platform was first made commercially available in 2015, its sequencing error rate was still very high (18). After several rounds of improvement, the sequencing error rate has reached the acceptable range (19, 20). This device is small enough to be held by one hand and can process one specimen at a time, making it particularly useful for routine use in clinical microbiology laboratories. The cost of the Oxford Nanopore Technologies' MinION device is inexpensive compared to the traditional NGS machines; and in our laboratory, the cost of sequencing one sample is ~90 USD. This is in fact more economical than sending the specimen out to private laboratories in our hospital setting. The turn-around-time for using the Oxford Nanopore Technologies' MinION device is <2 days, compared to 2–4 days if the sample is sent out. Furthermore, if the demand for the service is increased, the technology is also easily scalable. Such flexibility enables it to fit in clinical microbiology laboratories that handle different specimen volumes. All these advantages have made the Oxford Nanopore Technologies' MinION device an emerging technology platform in clinical microbiology laboratories.

## Data Availability Statement

The 16S rDNA gene sequence of Strain_HKU-SZH_1120 has been deposited in GenBank (Accession Number: OM095442).

## Ethics Statement

Written informed consent was obtained from the individual(s) for the publication of any potentially identifiable images or data included in this article.

## Author Contributions

FX and PW wrote the manuscript. FX reviewed the clinical data. SL supervised the microbiological investigations. YM, JI, and W-MC processed and analyzed the 16S rDNA gene sequence data. MZ and MG reviewed the pathological data. All authors have read and approved the final version of the manuscript.

## Funding

This study was partly supported by Sanming Project of Medicine in Shenzhen, China (SZSM201911014).

## Conflict of Interest

The authors declare that the research was conducted in the absence of any commercial or financial relationships that could be construed as a potential conflict of interest.

## Publisher's Note

All claims expressed in this article are solely those of the authors and do not necessarily represent those of their affiliated organizations, or those of the publisher, the editors and the reviewers. Any product that may be evaluated in this article, or claim that may be made by its manufacturer, is not guaranteed or endorsed by the publisher.
